# Reliability and Usefulness of Seedling Plant Data in Cassava Breeding

**DOI:** 10.3390/plants14172810

**Published:** 2025-09-08

**Authors:** Chalermpol Phumichai, Hernan Ceballos, Paphawe Pleeprom, Thanasak Chomsuk, Pasajee Kongsil, Wannasiri Wannarat, Wanwisa Siriwan, Thapakorn Jaisuwan, Marcelo Mollinari, Carlos Iglesias, Vichan Vichukit, Ed Sarobol, Chareinsuk Rojanaridpiched

**Affiliations:** 1 Department of Agronomy, Faculty of Agriculture, Kasetsart University, Bangkok 10900, Thailand; paphawe.pl@ku.th (P.P.); thanasak.cho@ku.th (T.C.); pasajee.k@ku.th (P.K.); wannasiri.w@ku.th (W.W.); vichukitvic@gmail.com (V.V.); agred@ku.ac.th (E.S.); agrcsr@yahoo.com (C.R.); 2 International Center for Tropical Agriculture (CIAT), Km 17, Recta Cali-Palmira Apartado Aéreo 6713, Cali 763537, Colombia; hernanceballosl54@gmail.com; 3 Department of Plant Pathology, Faculty of Agriculture, Kasetsart University, Bangkok 10900, Thailand; wanwisa.si@ku.th; 4 Doipui Research Station, Faculty of Agriculture, Kasetsart University, Chaingmai 50200, Thailand; thapakorn.ja@ku.ac.th; 5 Department of Horticultural Science, North Carolina State University, Raleigh, NC 27607, USA; mmollin@ncsu.edu (M.M.); caiglesi@ncsu.edu (C.I.)

**Keywords:** phenotypically estimated breeding values (PEBV), seedling plant information, unbiased populations, general combining ability, specific combining ability

## Abstract

Cassava breeding traditionally involves several years of phenotypic evaluation and field selection. The process begins with seedling evaluation trials (SETs), followed by single row trials (SRTs), and then progresses through preliminary, advanced and uniform yield trials. In recent years, significant efforts have been made to shorten the cassava breeding cycle through genomic selection, which relies on genotypically estimated breeding values (GEBVs). Breeders have typically performed visual selection during the SET stage, when each genotype in segregating populations is represented by a single plant. Despite the intense selection pressure applied at this stage—often eliminating 80–90% of genotypes—no data are collected prior to selection. As a result, breeders miss the opportunity to assess the degree and direction of dominance for key cassava traits, which remains largely unknown. This study provides pioneering insights based on seedling data collected over three years of field evaluations, along with the performance of selected genotypes at the SRT stage. Beyond its relevance for conventional inheritance studies, SET data can also be used to estimate phenotypically estimated breeding values (PEBVs) of progenitors, serving a similar purpose to GEBVs in genomic selection strategies. In fact, these two approaches to estimating breeding values can be highly complementary.

## 1. Introduction

In SE Asia, cassava is mostly used for the industrial production of starch [1,2], animal feed [3,4] and ethanol [5,6]. On the other hand, the direct consumption of cassava roots and leaves plays a critical role in food security in Africa [7,8]. Cassava is one of the most important crops in Thailand for domestic and export markets [9,10,11]. By 1975, Thailand emerged as the dominant world exporter of cassava chips, pellets and starches. In 1985 Thailand garnered 95% of the European cassava imports [12]. A key requirement for cassava development is linking farmers to markets [2]. Thailand is a prime example of linkages between farmers and markets that can contribute to the development of the cassava sector elsewhere.

Cassava’s resilience allows it to thrive on marginal soils with limited fertility, withstand adverse abiotic stresses, particularly water deficiency, and tolerate prevalent pest and diseases in Asia [13,14,15] and Africa [16,17]. However, the situation changed drastically in SE Asia with the introduction of the pink mealybug (quickly overcome through its biological control [18]) and Cassava Mosaic Disease (CMD).

CMD stands out as one of the most important biotic constraints for cassava production in Africa. The disease causes large yield losses that negatively impact millions of farmers [19,20]. Although CMD is present in India and Sri Lanka, it was absent in SE Asia until recently [21]. Unfortunately, the geminivirus responsible for CMD (Sri Lankan variant) was eventually reported, first in Cambodia [22], then quickly spreading to neighboring countries, including Vietnam, Thailand and China [23,24,25]. The spread of CMD in five major cassava-producing provinces of Thailand along the border with Cambodia has been surveyed. Prevalence of the disease was highest in Prachinburi (80%), followed by Sakaeo (43%), Buriram (37%), Surin (25%) and Sisaket (10%) provinces [24].

The most effective approach to the control of CMD is through the utilization of resistant cassava varieties and the production of disease-free planting material. A reliable source of resistance (CMD2), conferred by a single major gene, was identified and mapped in the 1990s [26]. Local cassava germplasm in SE Asia, which had never been exposed to the disease, generally lacked the levels of tolerance/resistance required to maintain the outstanding levels of productivity achieved before the appearance of the disease [27].

The economic impact of CMD in cassava productivity was first noticed in Cambodia. In the 2014–2016 period, cassava productivity reached a maximum of 23.8 t/ha, whereas a few years later (2018–2020) the average yield was only 21.5 t/ha. In Thailand productivity went down from 22.3 t/ha in 2014–2016 to 20.9 t/ha in the 2020–2023 period [28]. Impact of productivity could be detected earlier in Cambodia because the disease was first detected in that country.

Thailand introduced a source of resistance to CMD2 as early as 2013 (experimental clone C33). In 2018, five additional genotypes (TMS-IBA980505, TMS-IBA972205, TMS-IBA 920057, TMS-IBA 980581 and TMEB 419) carrying CMD2 were introduced from the International Institute of Tropical Agriculture (IITA-Ibadan). Recent phenotypic and genotypic screening of germplasm under disease pressure in SE Asia, however, has detected CMD2 in the local landrace Hanatee (named TAI9 in CIAT’s cassava germplasm collection), which is valued for its excellent cooking quality. In fact, Hanatee seems to be identical to other accessions of the germplasm collection (CR63 and PER262) and is also known with other names throughout SE Asia.

Cassava breeding relies on phenotypic recurrent selection carried out through several years of field evaluation from a single plant per genotype (Seedling Evaluation Trial-SET), through single-row (SRT), preliminary (PYT), advanced (AYT) and multilocation uniform (UYT) yield trials. In every case, plants are harvested typically 10–12 months after planting (MAP). The number of plants representing each genotype increases through the different stages as the total number of genotypes decreases. In recent years considerable efforts have been invested in assessing the breeding value of progenitors used by different cassava breeding projects through phenotypic [29,30] or genomic [31,32,33,34] approaches. Breeding value is a fundamental parameter that assesses the relative merit of progenitors based on the performance of the progenies they generate within a reference population.

Although a large proportion of clones are eliminated at the SET stage, there is limited information of the efficiency and predicting value of data coming from seedling plants. Similarly, there is very little information regarding the importance of maternal (e.g., cytoplasmic) effects for different traits in cassava. Therefore, the objective of this study was to assess the reliability of information generated at SETs and SRTs in crosses between sources of resistance to CMD and Thai elite varieties.

## 2. Results

### 2.1. Seedling Trials

Data from 4943 seedling plants were available (Table 1). All plants were grown in the field under experimental station conditions. The total number of evaluations conducted each year—607 in 2019, 3285 in 2020 and 1051 in 2021—illustrates the considerable imbalance in the dataset, a common feature in cassava breeding. Average yield in 2020 (3.32 kg/plant) was significantly higher than in 2021 (1.07 kg/plant), while results from 2019 were intermediate (2.31 kg/plant). Similarly, harvest index (HIN) values in 2019 and 2020 were considerably higher than those in 2021.

### 2.2. Maternal Effects

Maternal effects were assessed only when a minimum of ten seedling plants were available for the average of each cross direction. For the 2019 data, maternal effects could be evaluated for only one cross, 16 × 25 (Table 2). In contrast, eight and five F1 crosses met this criterion for the analysis of maternal effects in 2020 and 2021, respectively.

Maternal effects on standardized yield were significant in only one cross (13 × 25 in 2020) and were more frequently observed for above-ground biomass (AGB) and harvest index (HIN). However, it is important to note that the few significant cases of maternal effects may reflect field design artifacts rather than true genetic differences. For logistical reasons, SETs are planted with all progenies from a given cross (e.g., A × B) grouped together, while individuals from the reciprocal cross (e.g., B × A) are also planted together but typically in a different part of the field. As a result, maternal effects are completely confounded with environmental variation associated with the specific planting areas of each cross direction.

### 2.3. Breeding Values

**Figure 1:**
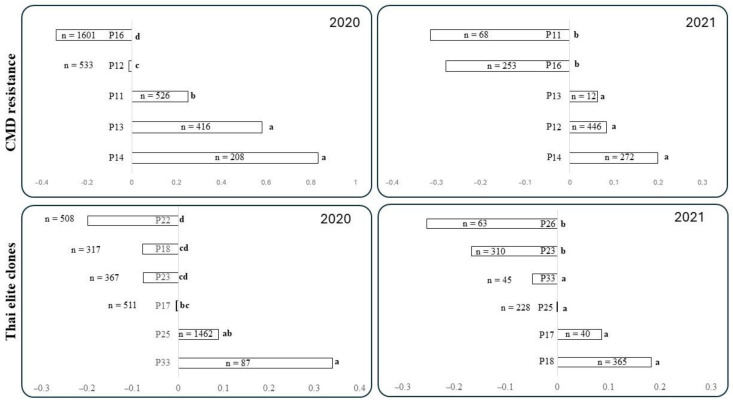
Breeding value for average standardized yield of progenitors involved in this study (those carrying CMD2 resistance on top and Thai elite clones at the bottom). Data from the seedling nurseries harvested in 2020 (**left**) and 2021 (**right**). Mean comparisons within each of the four plots were based on a *t*-test (unpaired observations and unequal variances). Averages with the same letter are not statistically different (*p* ≤ 0.01). Breeding value for Thai tester R11 was not included because of the small size of the half sib family (n = 33), which was only analyzed in 2020.

The averages for progenies from CMD-resistant lines and Thai elite testers are presented in Figure 1. Data from 2019 were excluded from the analysis because only one CMD-resistant line (C33) was involved in all crosses evaluated that year. The half-sib progeny from the Thai elite tester R11 was small (n = 33), consisting of three full-sib families evaluated only in 2020 (Table 2). Consequently, R11 was excluded from the breeding value analysis.

Figure 1 shows the averages of standardized yield for each half-sib family, with values indicating below-average (negative) or above-average (positive) performance. This information reflects the breeding value of each progenitor. Overall, there was good agreement between the results from 2020 and 2021. Among the CMD-resistant lines, P11 (IBA057) and P16 (C33) performed poorly, while progenies from P14 (IBA581) and P13 (IBA505) were outstanding; these differences were statistically significant. In fact, progenies from P14 ranked highest in both years. However, progenies from P12 (IBA205) showed contrasting performance, ranking second worst in 2020 but second best in 2021.

The breeding values for the Thai elite testers P17 (R5) and P25 (KU50) were consistently positive, while those for P23 (HB80) were mediocre in both years. Progenies from P18 (R9) exhibited strong genotype-by-environment (G × E) interaction, ranking best in 2021 but second worst in 2020. Similarly, the breeding values for P33 (HB100) were inconsistent between 2020 and 2021. The breeding value of P22 (HB60), which was evaluated only in 2020, was clearly negative.

Data presented in Figure 1 suggest that the breeding values of CMD-resistant lines were more consistent and influential than those of the Thai elite testers.

### 2.4. GCA and SCA in the Factorial Mating Design

Based on the results from the linear mixed model for yield (after log transformation), performance in 2021 was significantly lower (*p* < 2 × 10^−16^) than in 2020, with untransformed means of 1.042 kg/plant and 3.502 kg/plant, respectively. The G × E variance (0.449) was substantially higher than the genotypic variance (0.122), indicating considerable inconsistency in the performance of some crosses across years.

Regarding genetic effects, the progenitor lines carrying CMD resistance contributed meaningful variation (Lines GCA), whereas the variance among elite Thai progenitors (Testers GCA) was negligible, suggesting limited genetic differentiation for yield within this subset. GCA and SCA estimates are summarized in Table 3. The line-by-tester interaction (SCA) variance (*p* < 4.473 × 10^−7^) was substantial, indicating that specific cross combinations had a greater influence on yield than general parental performance alone. These results align with those from the fixed-effects ANOVA, which also revealed significant main effects and interactions. However, the mixed model framework offers more robust inferences given the unbalanced data structure and allows for partitioning variance into interpretable genetic components.

GCA values among the CMD-resistant lines differed significantly, with the best line progenitor being P14 (0.142) and the worst, as expected from Figure 1, P16 (−0.134). GCA values for P11 and P12 were intermediate. These results are fully consistent with those shown in Figure 1. Although the tester GCA variance was negligible compared to that of the lines, the rankings among the three testers also aligned with the breeding values in Figure 1, with KU50 exhibiting the highest GCA and HB80 the lowest.

In this dataset, non-additive effects (SCA) accounted for approximately 65% of the total genetic variation, while additive effects (GCA) explained around 35%. This indicates that yield differences were predominantly driven by specific parental combinations rather than by the general additive effects of individual parents (Table 3).

The crosses P14 = IBA581 × P18 = R9 and P14 = IBA581 × P25 = KU50 are particularly noteworthy. All three progenitors involved had positive GCA values and, interestingly, these crosses exhibited the highest SCA values. Both crosses share P14 as a progenitor. The consistent and outstanding performance of progenies from P14 suggests that this progenitor not only possesses excellent breeding value but may also display a promising heterotic response when crossed with Thai elite germplasm.

### 2.5. Single Row Trial

Only a small proportion of the genotypes evaluated as seedling plants advanced to single-row trials (526 out of 4943 genotypes). Selection at the seedling stage is generally visual and based on high-heritability traits such as erect plant architecture, as well as other important agronomic characteristics like HIN, FRY potential and adequate vigor. Extremely tall or weak plants are discarded. Consequently, the genotypes that reach the SRT stage represent a biased subset of the original populations.

Table 4 presents the average performance of progenies from CMD-resistant lines and Thai elite testers evaluated in the SRT across 2019, 2020 and 2021. The data from 2021 are particularly insightful, as they include four half-sib families from CMD-resistant lines and five from Thai elite testers. The best-performing tester was P25 (KU50) (Table 4), consistent with its breeding values in Figure 1 and GCA estimates in Table 3. Conversely, progenies from P23 (HB80) showed the lowest average performance in the SRT, aligning with its poorest GCA value among testers (Table 3) and mediocre breeding values in Figure 1.

The performance of progenies from the different CMD-resistant lines in the 2021 SRT generally agrees with data from the SET. The half-sib family from P16 (C33) showed the lowest FRY average (Table 4), consistent with its lowest GCA value (Table 3) and very poor breeding values (Figure 1). Progenies from P14 (IBA581) had the second highest FRY in the 2021 SRT. Although this was somewhat below expectations based on its GCA and breeding values (Table 3 and Figure 1), it was nonetheless excellent. In contrast, data from P12 (IBA205) were inconsistent, showing the highest FRY in the SRT but only intermediate GCA values and strong genotype-by-environment (G × E) interaction for breeding values in 2020 and 2021 (Figure 1).

SRT data from 2019 and 2020 are less informative than those from 2021 due to the limited number of half-sib progenies evaluated. In 2020, progenies from the CMD-resistant line P14 (IBA581) performed considerably better than those from P11 (IBA057), consistent with the data shown in Figure 1. Among the testers, progenies from KU50 (P25) exhibited slightly lower fresh root yield (FRY) than those from HB60 (P22) in 2019.

## 3. Discussion

This Discussion Section will address two key topics: first, the value of recording and analyzing data from the SET; and second, the specific insights gained from the different genotypes and crosses evaluated in this study.

### 3.1. The Usefulness of Estimating Phenotypically Estimated Breeding Values (PEBV)

Conventional cassava breeding begins with the SET, in which each genotype is represented by a single plant derived from germinated botanical seeds. Typically, all seedlings from the same female progenitor are planted consecutively in the field, followed by seedlings from a different female progenitor. As a result, there is a complete overlap between genetic variation (i.e., half-sib families) and environmental variation (i.e., specific sectors of the field where the half-sib families are planted). No formal field design is employed at this stage.

Breeders visually select the most promising seedling plants based on traits related to plant architecture, health, yield potential and overall vigor. In some cases, special traits such as amylose-free starch or high carotenoid content may also be considered [35,36]. Typically, only the selected genotypes are assigned a pedigree identification. Discarded genotypes are ignored, and neither their existence is recognized, nor their performance contributes to the breeder’s knowledge base.

This study aimed to assess the value of recording information at the SET stage as a useful approach for estimating phenotypically estimated breeding values (PEBVs), serving as an alternative or complement to genomic estimated breeding values (GEBVs). The data analyzed were influenced by environmental factors, which led, for example, to significantly lower average yields in 2021. Genotype-by-environment (G × E) interactions were also significant.

A major limitation of analyzing SET data is the inability to replicate individual seedling plants, as they have not yet been cloned. However, it is possible to replicate families by dividing each half-sib family into two or three groups of similar sizes and planting them as blocks in different parts of the field. Although the individual seedlings within each block differ genetically, the family effect would be replicated, enabling more detailed and robust analyses. A similar strategy has been successfully implemented at the SRT stage [30], but then segregating populations are already biased by the previous selection at the SET.

The limitations associated with how SETs are planted and the absence of a formal field design are partially mitigated by the fact that this study was conducted over three consecutive years, using different sets of genotypes derived from a common group of progenitors. As expected, there was no clear evidence of maternal effects, particularly with respect to root yield.

A second problem in cassava breeding is the common lack of balance in the number of seeds from different crosses and progenitors obtained from the crossing nurseries. This is exacerbated by the fact that the most desirable phenotype, from the agronomic point of view, is erect plant architecture which, by default, flowers late or not at all. Recent improvements manipulating flowering biology in cassava [37,38] are likely to make the production of seeds from different progenitors more homogeneous.

Averaging data across half-sib families to estimate PEBVs helps mitigate some of the challenges posed by the lack of a formal field design and has proven to be a valuable strategy. This approach requires recording data from large numbers of seedling plants—sometimes in the thousands—but the effort is justified. Collecting seeds from each crossing nursery takes up to two years, and growing a SET requires an additional year. Harvesting data from each seedling plant takes only 2–3 days and represents the only extra effort needed for the analyses described in this study. Importantly, this is the breeder’s only opportunity to analyze the original segregating populations prior to the intensive selection conducted at the SET stage. All subsequent selection stages, including the SRT, involve samples already biased by prior selection.

The availability of SET data would facilitate the estimation of the degree of dominance—if any -and the identification of the progenitor exerting it for various important traits in cassava. With few exceptions, such as resistance to whiteflies [39], cassava mosaic disease (CMD) [26], amylose-free starch [35] and carotenoid content [40], this essential information remains largely unavailable in cassava.

Interestingly, there is a strong concordance between the breeding values presented in Figure 1—based on the entire dataset (excluding data from P19)—and the results of the conventional GCA/SCA analysis shown in Table 3. Overall, there was also a reasonable level of agreement between data from the SET and SRT stages.

### 3.2. The Genetic Information Provided by PEBV

Excellent breeding values were consistently observed for the CMD-resistant lines P14 (IBA581) and P12 (IBA205), while P16 (C33) performed poorly overall, with its only positive feature being its role as a carrier of CMD2. Among the Thai elite germplasm, progenies from KU50 consistently exhibited above-average yield performance, whereas those from P23 (HB80) were consistently mediocre. Strong G × E, particularly year-by-genotype interactions, influenced the performance of other elite Thai progenitors. Overall, there was a reasonable level of agreement between SET and SRT data, suggesting that SET evaluations can be useful for predicting family performance once genotypes are cloned.

The GCA/SCA study further highlights the significant role of non-additive genetic effects (e.g., SCA) in the expression of complex traits such as FRY. These findings are consistent with previous phenotypic studies [30,40,41] and genomic analyses [31,32,33]. Consequently, breeding for complex traits like FRY is considerably more challenging than for traits governed by fewer loci or those with predominantly additive genetic effects, such as RSC [34].

Results from the GCA/SCA analysis for root yield showed significant GCA values for CMD-resistant lines compared to elite Thai testers. This finding is unexpected, as the Thai germplasm was anticipated to contribute strongly with yield-enhancing alleles. However, it was the introduced African germplasm that had a greater influence on yield. A plausible explanation is that years of breeding for high yield in Thailand have produced a more genetically homogeneous pool of progenitors, whereas the African germplasm may carry a more diverse set of alleles, leading to greater variation in their progenies. It is important to note that SETs were evaluated under conditions of low or negligible disease pressure.

P16 (C33) was the only non-bred progenitor among the CMD-resistant lines, and its progeny was by far the poorest performing half-sib family (Figure 1, Table 3). It is plausible to suggest that, as an unimproved progenitor, C33 still carries a substantial genetic load, which was passed on to its progeny, resulting in their poor performance.

Table 3 presents an interesting finding regarding P14. This progenitor not only had the highest GCA value, but two of its crosses also exhibited the highest SCA values. These outstanding SCA values in two full-sib families involving P14 suggest the possibility of a heterotic pattern between IBA581, of African origin, and the elite Thai germplasm included in this study.

## 4. Materials and Methods

Crosses among 13 parental cassava genotypes were made (Table 5). They included the released Thai varieties Rayong 5 (R5), Rayong 9 (R9), Rayong 11 (R11), Huay Bong 60 (HB60), Huay Bong 80 (HB80), Huay Bong 100 (HB100) and Kasetsart 50 (KU50) and the promising clone MKUC60-123-1 (MKUC). Thai genotypes were selected because of their high fresh root yield (FRY) potential, high starch content in the roots (RSC) and desirable plant architecture. However, except for KU50, which shows some degree of tolerance, they are susceptible to CMD.

The remaining five genotypes carry the CMD2 source of resistance to the disease in a heterozygous condition. One of these CMD-resistant genotypes (C33) came from Centro Internacional de Agricultura Tropical (CIAT) and is an experimental clone from the early efforts to develop a molecular map for the CMD2 locus and thus has not been bred for high productivity. The other four CMD-resistant varieties were IITA-TMS-IBA920057 (IBA057), IITA-TMS-IBA972205 (IBA205), IITA-TMS-IBA980505 (IBA505) and IITA-TMS-IBA980581 (IBA581) and came from the International Institute of Tropical Agriculture (IITA). These four clones not only carry CMD2 but also have been selected for their FRY and general agronomic performance in Nigeria. The progenitors involved, therefore, could be clearly split into CMD-resistant and non-resistant groups with five and eight genotypes, respectively.

### 4.1. Seedling Evaluation Trials (SET)

Controlled pollinations [44] were made between the Thai improved germplasm and the different sources of CMD resistance following an adaptation of the line x tester of principles originally described by Kempthorne [45]. Whenever possible, crosses were made in both directions (i.e., each progenitor was mated as both a male and a female) so direct and reciprocal versions of the same cross would be available (Table 6). The resulting crosses were evaluated at the seedling stage in trials conducted at the Tapioca Development Institute (TDI) between 2019 and 2021. By default, each genotype is represented by a single plant that is evaluated only once at the seedling stage. The populations of botanical seedlings (adding up to 4943 F1, Table 6) were generated at the TDI and Doi Pui Research Station, Kasetsart University, Thailand. In the SET, each family was planted with 1 × 1 m spacing between and within rows. For every nine seedlings, the HB90 check variety was planted.

Data for individual seedling genotypes at the SET were collected at harvest, 10 months after planting (MAP), for the following traits: fresh storage root yield (FRY) (kg plant^−1^), weight of above-ground biomass (AGB; kg plant^−1^) and harvest index (HIN), which was calculated as the total FRY divided by the total biomass (FRY + AGB). The plant type score (PTS) was assessed visually using a 1–5 scale, with 1 indicating a plant clearly better than average and 5 indicating a plant clearly worse than average.

#### 4.1.1. Maternal Effects and Breeding Values

Maternal effects were assessed in full-sib families where direct and reciprocal crosses were available (Table 5). The number of direct and reciprocal crosses were typically different (Table 6) and so were their respective variances. The significance of differences between means was therefore determined by using a *t*-test for unpaired observations with unequal variances. The standard deviation for *t*-tests was estimated as

s_d_ = √ [(s_1_^2^/n_1_) + (s_2_^2^/n_2_)]

where *s_d_* is the standard deviation used in the *t*-test, *s*_1_^2^ and *s*_2_^2^ are the variances of the first and second averages being compared, respectively, and *n*_1_ and *n*_2_ are the corresponding numbers of observations on which these averages are based.

The degrees of freedom for this *t*-test are calculated following Satterthwaite’s approach [46].

df = [(s_1_^2^/n_1_) + (s_2_^2^/n_2_)]^2^/{[(s_1_^2^/n_1_)^2^/(n_1_ − 1)] + [(s_2_^2^/n_2_)^2^/(n_2_ − 1)]}

#### 4.1.2. Standardizing Yield Data

Cassava breeding efforts often face the problem of unbalanced datasets representing different half- and full-sib families, as illustrated by data in Table 2. Since the number of progenies per family evaluated in different years varied considerably, it is difficult to assess the performance of families across years because of the strong G × E effects commonly observed in cassava research. To attenuate this problem, the analysis for yield potential (the variable most affected by G × E) was carried out using standardized values:

$$y_{\mathrm{st}}\;=\;(y_{i}-\;\bar{Y})/s$$

where *y_st_* and *y_i_* are, respectively, the standardized and untransformed values for yield, and $\bar{Y}$ and s are the average and standard deviation for the untransformed yield data, respectively.

#### 4.1.3. Assessing Breeding Values for Progenitors

All progenies from a given progenitor were pooled together as a half-sib family. As in the case of maternal effects, averages for each family were based on different numbers of observations, and their variances were generally non-homogeneous. Averages for half-sib families from each progenitor for y_st_ across years were used as a proxy for the estimation of breeding value. This approach, which follows Falconer’s definition for breeding value [29], offers robust information considering the large size of the progenies derived from each progenitor (Table 6).

The significance of comparisons between averages was assessed through *t*-tests for unpaired observations with unequal variances and using Satterthwaite’s approach (as described above for the study of maternal effects).

#### 4.1.4. GCA and SCA

A common problem in cassava breeding is the large variation in seeds obtained from the crossing nurseries (as illustrated by data in Table 6). Most genetic designs based on the analysis of full- and half-sib families rely on a balanced set of crosses, a requirement that is obviously not met by the full dataset presented in Table 2. Therefore, a subset of materials that offer an acceptable level of balance was selected for an assessment of GCA and SCA effects.

The selected subset of families from the SET was evaluated in the years 2020 and 2021. The individuals from the same cross evaluated in both years were obviously different samples from the same family and thus allowed for a replicated effect. Both direct and reciprocal crosses were pooled together. This subset of families allowed for a factorial mating analysis similar to the line x tester design [45]. Four CMD-resistant ‘lines’ and three CMD-susceptible (elite Thai varieties) ‘testers’ were used to estimate main (additive) and interaction (non-additive) effects. In a breeding context, these can be thought of as “GCA-like” (lines and testers) and “SCA-like” (line × tester). For the genotype × environment (G × E) analysis, each cross is treated as a genotype, and Year (2020 vs. 2021) is treated as the environment [29,47,48,49].

Several models were evaluated for data analysis. Initially, a fixed-effects factorial ANOVA of the untransformed data for yield was conducted. However, diagnostic plots of residuals indicated non-normal and heteroscedastic patterns. Eventually, a mixed-effects model of the log-transformed data for yield complied with the required assumptions for statistical analysis. The model allowed for a robust estimation of variance components (e.g., GCA, SCA) and the generation of best linear unbiased predictions (BLUPs), while appropriately modeling the data’s hierarchical and crossed structure. Residuals were more symmetric and homoscedastic on the log scale, and no glaring random-effects normality violation was detected. Therefore, the log transformation was used for the final factorial mating model.

### 4.2. Single Row Trial (SRT)

For the SRT, data were collected 10 MAP. The following traits were measured: FRY (expressed in t ha^−1^), HI, PTS, starch content in the roots (RSC), measured using the gravimetric method described by Kawano [50] and expressed as a percentage, and the average number of roots per plot (NRP).

The best seedling plants were selected, cloned and evaluated in a clonal evaluation trial (SRT). Each genotype was planted in a single row with five plants, spaced 1 × 1 m between and within rows. The trial followed an augmented block design. A total of 526 genotypes were evaluated from 2019 to 2021, across two locations—the Tapioca Development Institute (TDI), located in Huay Bong, Dan Khun Thot District, Nakhon Ratchasima, Thailand (15.1831° N, 101.4566° E), and the National Corn and Sorghum Research Center (Suwan Farm), Kasetsart University, Nakhon Ratchasima Province, Thailand (14.6422° N, 101.3152° E). Two check varieties, HB90 and HB100, were included for comparison. Each genotype was evaluated only once at the SET and at the SRT the following year.

Both TDI and Suwan Farm were locations with low disease pressure at the time the field evaluations were conducted.

## 5. Conclusions

This study demonstrates that the additional costs associated with generating and analyzing SET data in cassava are clearly justified. Data from unselected (and therefore unbiased) segregating populations derived from biparental crosses provide critical insights into the degree and direction of dominance—if any—for key traits in cassava, a type of information that remains largely unavailable in this crop. Furthermore, beyond conventional inheritance studies, SET data can be used to estimate the breeding value of progenitors (e.g., PEBV), serving a similar purpose to GEBV in genomic selection strategies. In fact, these two approaches to estimating breeding values can be highly complementary. However, the relevance of breeding values varies considerably depending on the relative importance of non-additive effects in the expression of a given trait. While they may be less reliable for predicting fresh root yield (FRY), they can be highly informative for traits such as plant architecture, resistance to pests and diseases, harvest index (HIN) and, if available, root starch content (RSC).

## Figures and Tables

**Table 1:** Average and standard deviation values for seedling trials in 2019, 2020 and 2021.

Year	n	FRY		AGB		HIN	
		Average	St. Dev.	Average	St. Dev.	Average	St. Dev.
2019	607	2.309	1.581	1.941	1.589	0.539	0.120
2020	3285	3.320	2.203	2.317	1.907	0.595	0.095
2021	1051	1.067	0.787	1.111	0.819	0.483	0.160

**Table 2:** Mean comparison (*t*-test, unpaired observations and unequal variances) between direct and reciprocal crosses for yield, stem and harvest index. Comparisons were made only when averages were based on at least ten seedling plants per direct or reciprocal cross. The df information (separated by a slash) relates to the Line × Tester and then the Tester × Line direction of the respective F1 cross, * and ** indicate statistical significance at *p* < 0.05 and *p* < 0.01, respectively.

Cross	df	Yield		*t*-Test	AGB		*t*-Test	HI		*t*-Test
		A × B	B × A		A × B	B × A		A × B	B × A	
**Year 2019**										
16 × 25	195/188	2.23	2.36	−0.78	2.18	2.04	0.76	0.49	0.54	−3.74 **
**Year 2020**										
12 × 17	11/88	2.98	2.61	0.78	1.91	1.82	0.33	0.60	0.59	0.48
12 × 25	48/259	3.39	3.50	−0.35	2.37	2.34	0.11	0.59	0.61	−1.87
13 × 25	13/164	2.23	5.17	−7.02 **	1.20	3.15	−8.42 **	0.64	0.64	0.03
16 × 17	67/85	2.35	2.40	−0.18	1.44	1.80	−2.06 *	0.59	0.57	0.94
16 × 23	43/115	2.48	2.63	−0.55	1.64	1.68	−0.18	0.60	0.62	−1.37
16 × 25	280/310	2.31	2.46	−1.27	1.87	2.01	−1.07	0.57	0.56	1.04
16 × 22	429/42	2.92	2.79	0.58	2.11	2.10	0.10	0.58	0.57	0.24
13 × 23	19/11	3.98	3.06	0.94	2.55	1.16	1.84	0.61	0.72	−2.77 **
**Year 2021**										
12 × 23	59/64	0.93	0.90	0.20	1.41	0.69	5.56 **	0.41	0.55	−4.69 **
12 × 18	143/15	0.97	1.40	−1.89	0.76	1.17	−3.16 **	0.54	0.54	−0.18
14 × 18	50/22	1.75	1.74	0.04	1.23	1.67	1.83	0.57	0.50	2.25 *
14 × 23	74/13	0.81	0.90	−0.57	1.09	1.40	−1.25	0.44	0.39	1.20
12 × 25	55/34	1.18	1.08	0.61	0.82	1.33	−3.52 **	0.59	0.46	5.63 **

**Table 3:** General and specific combining ability values of FRY from the factorial mating design. Within parenthesis are the GCA values for the tester and line progenitors. SCA values are presented in the body of the table.

		Tester		
		18 = R9	23 = HB80	25 = KU50
Line	GCA	(2.006 × 10^−7^)	(−9.594 × 10^−7^)	(7.588 × 10^−7^)
11 = IBA057	(−0.025)	−0.114	−0.115	0.183
12 = IBA205	(0.016)	0.066	−0.070	0.035
14 = IBA581	(0.142)	0.284	−0.274	0.256
16 = C33	(−0.134)	−0.150	0.044	−0.146

**Table 4:** Breeding values for different variables measured in single-row trials of materials selected at the seedling stage. Within each category, progenitors are ordered from the lowest to the highest FRY average.

Progenitor	n	Root Number	AGB	FRY	RSC	HIN
**Year 2019**						
16 (C33)	72	36.26	9.28	14.43	25.86	0.61
25 (KU50)	42	38.51	8.79	14.26	26.77	0.61
22 (HB60)	30	33.12	9.96	14.67	23.95	0.60
**Year 2020**						
11(IBA057)	7	8.89	4.90	1.33	NA	0.20
14 (IBA581)	9	18.54	5.88	4.99	NA	0.38
25 (KU50)	16	14.32	5.45	3.39	NA	0.30
**Year 2021**						
16 (C33)	61	NA	22.19	13.24	19.16	0.39
11(IBA057)	22	65.30	18.78	15.78	22.77	0.47
14 (IBA581)	171	66.48	20.96	16.28	19.12	0.44
12 (IBA205)	184	53.31	18.50	20.44	21.12	0.54
23 (HB80)	173	56.82	17.62	16.66	18.97	0.49
18 (R9)	132	63.24	24.28	17.19	20.50	0.42
26 (MKUC)	8	NA	24.22	17.23	20.89	0.40
33 (HB100)	24	42.12	12.25	18.99	20.44	0.58
25 (KU50)	101	69.33	19.93	20.27	21.65	0.51

**Table 5:** List of progenitors used in the study.

ID	Progenitor (Code)	Source	Germplasm Description
**Sources of resistance to CMD**			
11	IITA-TMS-IBA920057 (IBA057) *	IITA	Elite clone
12	IITA-TMS-IBA972205 (IBA205) *	IITA	Elite clone
13	IITA-TMS-IBA980505 (IBA505) *	IITA	Elite clone
14	IITA-TMS-IBA980581 (IBA581) *	IITA	Elite clone
16	C33 (C33) *	CIAT	Experimental clone
**Elite, CMD-susceptible, Thai genotypes**			
17	Rayong 5 (R5)	RYFCRC	High yield released variety
18	Rayong 9 (R9)	RYFCRC	High yield released variety
19	Rayong11 (R11)	RYFCRC	High yield released variety
22	Huay Bong 60 (HB60)	TTDI	High yield released variety
23	Huay Bong 80 (HB80)	TTDI	High yield released variety
25	Kasetsart 50 (KU50)	KU	High yield released variety
26	MKUC123-1 (MKUC)	KU	High yield elite clone
33	Huay Bong 100 (HB100)	TTDI	High yield released variety

IITA: International Institute of Tropical Agriculture, CIAT Centro Internacional de Agricultura Tropical, RYFCRC: Rayong Field Crops Research Center, TTDI: Thai Tapioca Development Institute, KU: Kasetsart University. * CMD2-linked SNP markers, S12_7926132, S12_7926163 and S14_4626854, located on chromosomes 12 and 14 and designed by Dr. Ismail Rabbi [42,43]. For marker validation, young leaf tissues from these clones were submitted to Intertek AgriTech for low-density SNP genotyping, following the standard protocol established by the CGIAR Excellence in Breeding (EiB) platform (https://excellenceinbreeding.org/toolbox/services/low-density-genotyping-service, accessed on 24 September 2021). CIMMYT-CGIAR services for the support on genotyping.

**Table 6:** Number of seedling plants evaluated over three years (2019, 2020 and 2021) of field trials. When both direct and reciprocal crosses were available, two numbers (separated by a slash) are shown within a cell. Progenitors listed in the columns carry the source of resistance to CMD, while progenitors in the rows represent elite Thai varieties.

Year	Progenitor	11	12	13	14	16	Total	
2019								
2020	17	79	9/90	3/120	58	65/87	511	551
2021			1/31		3	5	40	
2019								
2020	18	22	35	46		214	317	682
2021		16	143/15	2	50/22	6/111	365	
2019								
2020	19		4	12		16/1	33	33
2021								
2019						166	166	
2020	22		9	28		427/44	508	674
2021								
2019						21	21	
2020	23	116	54	11/19	9	45/113	367	698
2021		36	59/64		75/12	64	310	
2019						383	383	
2020	25	272	48/259	13/164	116	312/278	1462	2073
2021		7	55/34	5	8/63	56	228	
2019						37	37	
2020	26							100
2021		9	26	5	19	4	63	
2019								
2020	33	37	25		25		87	132
2021			18		20	7	45	
2019						607		607
2020		526	533	416	208	1602		3285
2021		68	446	12	272	253		1051
Total		594	979	428	480	2462		4943

## Data Availability

The data that support the results of this study are available from the corresponding author.

## Abbreviations

The following abbreviations are used in this manuscript:

FRY	Fresh storage root yield (kg plant^−1^)
AGB	Above-ground biomass (kg plant^−1^)
HIN	Harvest index
MAP	Month after plant
PTS	Plant type
RSC	Starch content in the roots
NRP	Average number of roots per plot
SET	Seedling evaluation trials
SRT	Single row trial
PEBV	Phenotypically estimated breeding values
GEBV	Genotypically estimated breeding values
